# Supramolecular Organization in Monolayers of Pyrene-Functionalized Asymmetric Twist-Bend Nematic Dimers: Comparison with a Three-Dimensional Crystal Structure

**DOI:** 10.1021/acs.langmuir.5c00551

**Published:** 2025-04-02

**Authors:** Agnieszka Maranda-Niedbała, Tomasz Jaroch, Rebecca Walker, Piotr Pięta, Ewa Górecka, Corrie T Imrie, John MD Storey, Adam Proń, Robert Nowakowski

**Affiliations:** 1 Institute of Physical Chemistry, Polish Academy of Sciences, Kasprzaka 44/52, Warszawa 01-224, Poland; 2 Faculty of Chemistry, Warsaw University of Technology, Noakowskiego 3, Warszawa 00-664, Poland; 3 Department of Chemistry, University of Aberdeen, UK3G28 Meston Building, Old Aberdeen Campus, Meston Walk, Aberdeen AB24 3UE, U.K.; 4 Department of Chemistry, University of Warsaw, Żwirki i Wigury 101, Warszawa 02-089, Poland

## Abstract

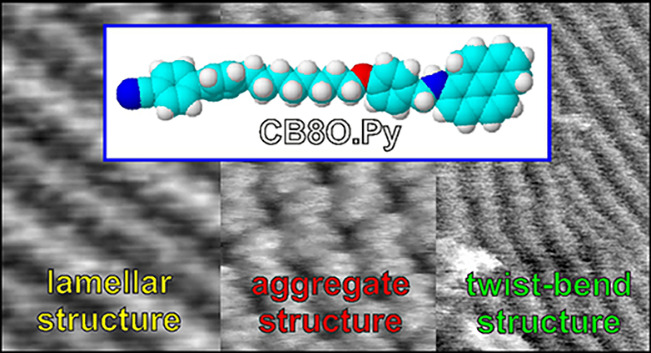

Two-dimensional (2D) supramolecular organization of asymmetric liquid crystal dimers containing two chemically different mesogenic groups, namely, cyanobiphenyl and pyreneiminomethyl phenoxy groups, linked with an oligomethylene linker of different length (six or eight −CH_2_– units), was studied using scanning tunneling microscopy. For this purpose, monolayers of these dimers were deposited on an HOPG substrate from their solutions in hexane by drop-casting and imaged at nanometer resolution after drying. A detailed discussion of two possible different supramolecular organizations is presented. In the case of the first one, termed the “lamellar structure”, the molecules in each layer are arranged in parallel molecular rows. Comparative analysis of the monolayer structure with already reported XRD data on 3D organization of the same molecules in a single crystal revealed significant differences resulting from (i) different ordering preferences in 2D and 3D systems and (ii) effects of the interactions of the deposited molecules with the graphite substrate. A small extension of the linker by two methylene groups does not change the type of ordering in the monolayer but causes an extension of the unit cell in one direction, indicating that the lamellar structure is stabilized by rows of densely packed large aromatic pyrene units. Studies of the derivative with a longer linker (containing eight −CH_2_– units) also showed the possibility of the occurrence of an alternative type of its monolayer organization, termed here “aggregate structure”. In this case, the monolayer consists of ordered aggregates of two molecules. According to the authors’ knowledge, this type of structural organization has not yet been reported for this family of compounds. It is, however, qualitatively consistent with the already described supramolecular ordering in CBO5O.Py (CCDC 2019485), a compound closely resembling the dimers studied in this research.

## Introduction

Liquid crystal dimers make up an interesting group of compounds exhibiting unique supramolecular properties. For this reason, they have been a subject of intensive research in a number of scientific centers. Progress in this domain of soft matter chemistry and physics has been successively reported in several review articles and books.^1−14^ By definition, these molecules consist of two mesogenic units connected by a flexible linker, most often an alkyl or alkoxy chain. Specific properties of these compounds include, among others, a strong dependence of their supramolecular properties on the length of the linker. This dependence is well-illustrated by the characteristic parity effect, which manifests itself in a significantly different type of ordering depending on either the even or odd number of structural units in the linker, e.g., methylene groups in the case of an alkylene linker. The above-mentioned differences in the supramolecular arrangement are associated with a different shape of the dimer containing an odd-membered or even-membered linker inducing different distributions of intermolecular interactions in the layer. Another feature that makes this type of compound very attractive is the ability of these nonchiral molecules to form a chiral nematic twist-bend phase. This unique organization, independently predicted theoretically by Meyer^15^ and Dozov,^16^ was experimentally confirmed in 2011 by Cestari et al.^17^ The synthesis of appropriate dimers that form twist-bend phases still remains a challenging task. Detailed investigations of these liquid crystals are not only of interest for fundamental research, mainly related to the mechanism of spontaneous mirror symmetry breaking, but also open the possibility to apply them as components of optical or optoelectronic devices such as fast elementary optical devices,^18^ memory devices,^19−21^ and lasers of smoothly tunable emission wavelengths in the visible-light range.^22^ A model example of molecules exhibiting the above features is symmetrical cyanobiphenyl dimers. They show a strong parity effect, being capable of forming a nematic twist-bend phase in the case of derivatives with odd-membered linkers. The twist-bend nematic phase is characterized by a lack of the molecules’ positional order, similar to “classical” nematics. However, in this phase, the molecules spontaneously form a heliconical structure, in which molecules are inclined at an arbitrary angle (<90°) to the helix axis. The helical pitch in the twist-bend nematic phase is exceptionally short, spanning only over few molecular distances, while the conical angle is 20–30°. This phase shows a markedly different structure from the nematic structures observed in chiral molecules, where the helical pitch is perpendicular to the molecular long axis and typically spans over hundreds of nanometers. In particular, the presence of this phase was experimentally verified for a dimer of this family, namely, CB7CB, in which two cyanobiphenyl units are connected with a chain containing seven methylene groups.^23^

More recently, asymmetric dimers, or more broadly asymmetric oligomers, in which mesogenic units are chemically different, emerged as a new family of liquid crystals.^24−28^ In this case, the presence of two distinctly different mesogenic units gives rise to a larger diversity of intermolecular interactions. Smectic phases, diversifying their structure via varying degrees of overlapping of their mesogenic units or their intercalation, can be given here as instructive examples.^13^ It is obvious that the synthesis of appropriately designed asymmetric molecules may lead to liquid crystals that, under specific conditions, form diversified supramolecular aggregations, including the above-mentioned twist-bend nematic phase.

The basic research tool used in the research reported here is scanning tunneling microscopy (STM), which enables imaging and local environment studies of various types of solid surfaces at nanometric resolutions. These possibilities fit well with the needs of precise observation of the frequently complex supramolecular organization of liquid crystals. In addition, appropriately performed STM investigations may reveal the effects of interactions between various particular segments of the molecules on the resulting supramolecular organization in monolayers deposited in suitable substrates (see for example recent papers of our group^29−32^).

It should be emphasized that STM is basically dedicated to studies of surfaces of conducting materials showing uniform surface electrical conductivity. In the case of monolayers of organic electroactive materials, the surficial conductivity is not homogeneous and depends on the distribution of the structural segments transporting the tunneling current. It should be noted that the first successful organic monolayer imaging with molecular resolution was achieved for low-molecular-mass liquid crystals and in particular for the two-dimensional arrangement of alkyl derivatives of cyanobiphenyl.^33−42^ Based on the presented STM images, a number of issues were then discussed, e.g., (i) the effect of the length of the alkyl substituent and the parity on the resulting supramolecular organization, (ii) polymorphism revealed as a difference in the 2D and 3D unit cell parameters, (iii) the effect of the substrate on the monolayer structural organization, and (iv) ordering of mixed systems consisting of cyanobiphenyl derivatives with different lengths of the alkyl substituent. It is important to add that these were pioneering works for the development of scanning tunneling microscopy. For the first time, important questions were raised concerning the STM image contrast mechanisms when this technique is applied to surfaces with locally inhomogeneous electrical conductivity. Thus, it opened up new applications of STM involving electrically and electronically complex surface imaging (e.g., thin films of organic semiconductors).

To the best of our knowledge, our previous paper^43^ described for the first time the extension of the molecular resolution STM imaging to liquid crystal dimers. This work focused on self-assembly of symmetric cyanobiphenyl dimers on the HOPG. Visualization of the layer surfaces obtained for a series of homologues with different linker lengths enabled a detailed analysis of the supramolecular ordering, including (i) the influence of the linker length, (ii) the parity effect, and (iii) the formation of a chiral structure. A particularly important result presented was the first images of the twist-bend nematic phase at submolecular resolution. Understanding the STM images and analysis of the locally complex structure of this phase were made possible by the correlation of the STM-derived 2D structures with the 3D organizations in single crystals determined by XRD.

The supramolecular ordering of liquid crystals is a consequence of a competition between the tendencies to adopt either crystalline or glassy forms. The majority of low-molecular-mass liquid crystals exhibit low glass transition temperatures (*T*_g_), far below room temperature. An increase in the *T*_g_ value can be achieved by introducing larger rigid aromatic units. Previous investigations of compounds containing naphthalene derivatives confirmed the validity of this approach.^44^ Following this strategy, in this work, we focused on a family of asymmetric dimers consisting of the spatially more extended pyrene moiety attached to the cyanobiphenyl group through a flexible linker. In the subsequent text, these compounds are abbreviated as CB*n*O.Py, where *n* indicates the number of methylene groups in the linker. Therefore, these compounds should combine traditional liquid crystalline properties associated with the presence of the cyanobiphenyl unit with the glassy behavior induced by a large aromatic unit such as pyrene. It should be noted that both symmetric and asymmetric liquid crystal dimers containing pyrene have already been reported.^45−56^ XRD measurements, carried out also for compounds investigated in this research, showed that the temperature of the glassy twist-bend nematic phase formation varies in the range 40–80 °C, depending on the linker length. Thus, in all cases, it significantly exceeds room temperature, although the process usually requires high quenching rates. The corresponding temperature in the case of the above-mentioned symmetric cyanobiphenyl dimer (CB7CB) is of the order of ∼4 °C, confirming the expected effect of the pyrene unit introduction.^23^

Here, we report detailed scanning tunneling microscopy investigations carried out at high-resolution with the goal of precisely following the ordering of CB*n*O.Py dimers in monolayers formed on a pyrolytic graphite substrate. Microscopic images obtained for two derivatives differing in the aromatic linker length allowed us to propose the pattern of the supramolecular ordering and to analyze the influence of the linker length on the resulting 2D supramolecular structure. It is important to note that the prediction of supramolecular ordering in the case of the studied dimers is difficult due to the presence of two chemically different mesogenic units attached to an aliphatic linker. Complex assembly of such molecules gives rise to a variety of intermolecular interactions between differently located molecular segments. In particular, the presence of fused pyrene units of aromatic nature introduces the possibility of π–π stacking interactions in the supramolecular aggregation. Thus, new questions must arise concerning the effect of these interactions on the crystalline vs glassy states of these materials. This knowledge is also fundamental for elucidating various physicochemical properties of the layers formed. Another important point of the research presented here is the correlation between the 2D structural organization in the monolayer, determined on the basis of STM, and the 3D one derived from the XRD data, the latter being already deposited in the crystallographic database.

## Experimental Section

### Synthesis

The studied liquid crystalline dimers (CB*n*O.Py, *n* = 6 and 8) were prepared through a multistep procedure, described in detail in the Supporting Information of ref (54) together with analytical data.

### Crystal Structures (Scanning Tunneling Microscopy)

The monolayers were deposited by drop-casting saturated solutions of the two studied dimers in hexane on a freshly cleaved surface of highly oriented pyrolytic graphite (SPI Supplies, USA). After evaporation of the solvent at room temperature, the samples were imaged in nanometric resolution using a scanning tunneling microscope (STM system manufactured by the group of Prof. K. Wandelt at the University of Bonn, Germany).^57^ Prior to performing studies, the STM scanner was calibrated using a typical HOPG surface imaging procedure. Imaging of the samples was performed at ambient conditions in constant current mode using mechanically cut Pt/Ir tips (80/20%). For each dimer, the imaging was performed multiple times on different scanning areas and for several samples. The proposed models of the 2D supramolecular structure were obtained by correlating the topology of the surface deduced from STM images and the molecular model of the investigated dimers.

### Twist-Bend Nematic Phase (Atomic Force Microscopy)

The preparation of samples for twist-bend nematic phase investigations required a different procedure. First, the dimers were directly deposited on an atomically flat substrate surface (mica or HOPG). Then, the samples were heated until the deposited matter melted, and subsequently, they were quickly introduced into liquid nitrogen. The microscopic investigations were performed after the samples were removed to ambient conditions, and their temperature was stabilized at the RT level. A commercial tandem system, Icon (Bruker, USA) installed in an MB200B glovebox (MBraun, USA), was used. Imaging was carried out in an inert atmosphere (Ar, N5.0) in the “tapping” mode using standard ScanAsyst-Air-HD AFM probes (Bruker, USA).

### DFT Calculations

The DFT calculations were performed using the Gaussian16 Revision C.01^58^ package and employing the hybrid B3LYP^59−61^ exchange correlation potential combined with the 6-31G(d,p) basis set. Ground-state geometries were fully optimized for all investigated molecules in vacuum. For this purpose, due to a rather ambiguous “direction” of the oligomethylene linker, appropriate energy calculations were performed for different linker geometries. The obtained results indicated that the dihedral angle between the plane of the linker carbons and the plane of the benzene ring connected the imine nitrogen atom can adopt two values, i.e., either close to 0° or +180°. Its effect on the energy of the molecule is, however, very small.

## Results and Discussion

### Molecular Modeling and Dimensions of the Molecule

**Figure 1:**
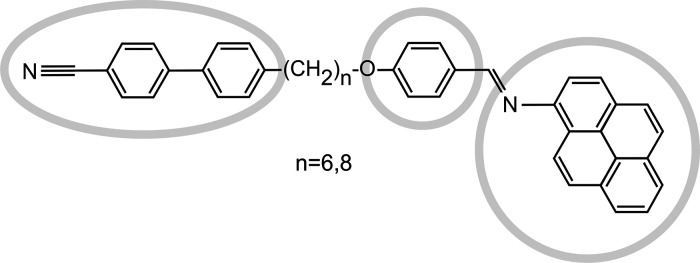
Chemical structure of the investigated molecules 4′-[ω-(4-{(*E*)-[(pyren-1-yl)imino]methyl}phenoxy)alkyl][1,1′-biphenyl]-4-carbonitriles (CBnO.Py, *n* = 6 and 8). The gray circles and ellipse mark different conductive segments of the molecules. In the STM images, they are also shown for clarity.

The investigated molecules consist of two different mesogenic units, namely, cyanobiphenyl and pyreneiminomethyl phenoxy groups connected with an oligomethylene linker of different lengths (six or eight −CH_2_– units) (Figure 1). Modeling the free molecule in a gas phase in its most extended conformation indicates that the molecular width is significantly different on both sides of the molecule and corresponds to the width of the mesogenic group, i.e., 0.5 and 0.73 nm for the cyanobiphenyl unit and the pyrene one, respectively (determined as the largest distance between hydrogens in the phenyl ring and the longest distance between hydrogen atoms located perpendicularly to the longitudinal axis of pyrene). The length of molecules varies with the linker and corresponds to 3.33 and 3.59 nm for CB6O.Py and CB8O.Py, respectively (measured as the distance between the nitrogen atom of cyanobiphenyl and the outermost hydrogen atom of the pyrene group). DFT calculations of the dimer’s electronic structure indicate that both mesogenic units are effectively separated by the alkylene linker, making them electronically independent.

### Structure A (Lamellar Structure)

**Figure 2:**
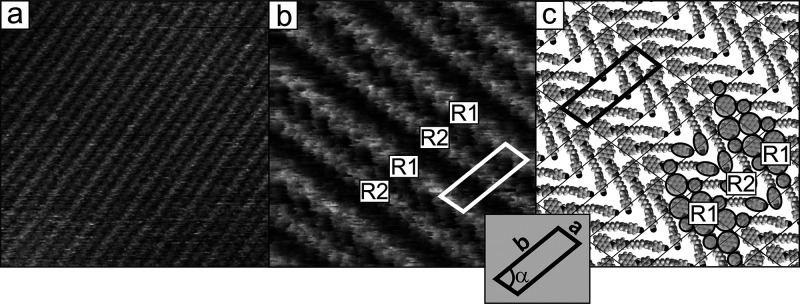
(a, b) STM images and (c) corresponding model of adsorption geometry of the CB6O.Py monolayer on HOPG (structure A). Scanning area and parameters: (a) 46 × 46 nm^2^, Δ*z*_max_ = 0.72 nm, *I_t_* = 1 nA, and *V*_tip_ = −0.85 V; (b) 15 × 15 nm^2^, Δ*z*_max_ = 0.48 nm, *I_t_* = 1 nA, and *V*_tip_ = 0.8 V.

Figure 2a,b shows representative STM images of the surface of the CB6O.Py monolayer formed on a HOPG substrate. The regular structure of the monolayer surface indicates that it consists of large areas of uniformly ordered molecules. This observation confirms the strong tendency of this adsorbate to self-organize. The existence of relatively large ordered domains may imply that the formation of 2D supramolecularly ordered aggregations is thermodynamic rather than kinetically controlled. In STM images, the monolayer appears as a set of well-resolved bright parallel stripes indicating ordering of this adsorbate into molecular rows. At this point, it should be recalled that the image contrast in STM is a combination of two factors, namely, (i) real topology, which makes higher areas imaged as brighter, and (ii) the electronic effect related in practice to the local variability of the electron density or electron work function. The structures of the investigated dimers indicate that the image of a single molecule in submolecular resolution should contain at least two separated bright areas originating from the presence of the mesogenic units. These regions of the molecule are brighter, compared to those corresponding to the methylene linker, because they are geometrically larger and characterized by higher electron density and electrical conductivity. A similar effect was observed in STM images of previously studied monolayers of alkyl derivatives of cyanobiphenyl and their symmetric dimers.^43^ This is a general phenomenon in these types of molecules where the mesogenic groups dominate the STM images, rendering the alkyl/alkylene groups virtually invisible. However, in the case of asymmetric dimers, described here, it must be remembered that both mesogenic units can yield images of different contrast due to their different chemical structure. The bright parallel stripes observed on the surface of the monolayer should therefore be associated with areas of ordered mesogenic units separated by darker areas corresponding to methylene linkers, which are essentially electrically inactive. Observations carried out with higher resolution (Figure 2b) provide more detailed information. The first characteristic feature of the monolayer surface is the fact that each two successive bright stripes are not equivalent. These dissimilarities are visible both in low-resolution images of large surface areas (Figure 2a) as different brightnesses and in higher-resolution images (Figure 2b) in the form of clearly visible differences in the internal structure of the neighboring stripes (marked R1 and R2, respectively). The second characteristic feature concerns the distance between the bright stripes. This distance between a given stripe and its two closest neighbors slightly differs. As seen in Figure 2b, the stripes are, in consequence, organized in pairs; the distance between them within the pair is shorter than the distance between the pairs. This clearly visible periodicity of the internal structures of the bright stripes, in particular the R1 ones, made possible the determination of the unit cell of ordered molecules in the monolayer (marked with a rectangle in Figure 2b). The unit cell parameters estimated from the STM image are as follows (*a*, *b*, and α): 1.45 ± 0.1 nm, 4.5 ± 0.15 nm, and 75 ± 2°. It should be noted that the larger dimension of the unit cell, 4.5 nm (along the (*b*) axis), is significantly larger than the length of the CB6O.Py dimer in its most extended conformation (3.3 nm). The above fact, together with the differences in the brightness described above and the internal structure of the adjacent bright stripes, indicates that the molecules in the monolayer are arranged in rows. However, a characteristic feature of this organization lies in the different orientations of the molecules in two subsequent rows. This is caused by the natural tendency of the mesogenic groups to interact with groups of the same type. In consequence, this leads to a herringbone-type ordering shown in Figure 2c. The proposed molecular arrangements in the layer explain the observed differences in the adjacent bright stripes, implying that they correspond to the areas of chemically different mesogenic groups. The much brighter stripe, marked by R1 in the STM image, reflects the location of the mesogens containing pyrene and is characterized by a more dense and complex arrangement of these mesogenic units. In comparison, the stripe marked as R2 exhibits lower brightness in the STM image, and this can be ascribed to a less extended aromatic system of supramolecularly interacting cyanobiphenyl units.

**Figure 3:**
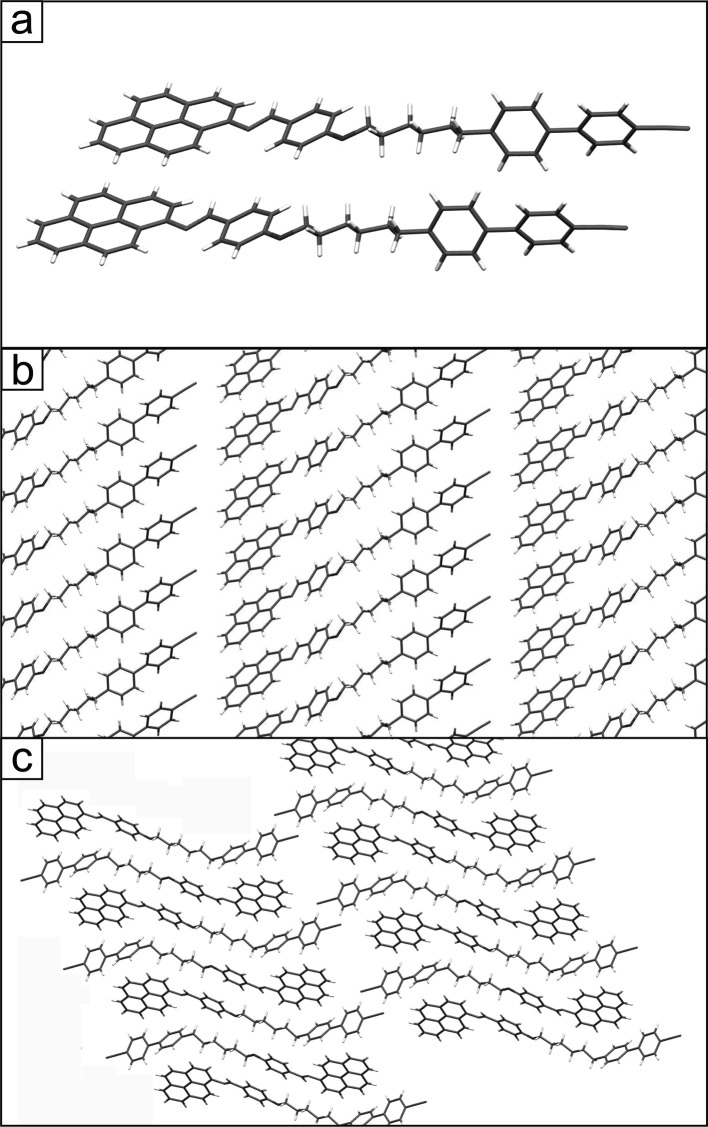
Crystal packing of CB6O.Py in the α polymorph (a, b) in the (010) plane (molecules are oriented parallel) and (c) in the (100) plane (from crystallographic database CCDC no. 2019482).

At this point, an important question arises concerning the correlation of the observed structure of the CB6O.Py monolayer on HOPG with the organization of the same molecule in the 3D single crystal. XRD studies of the crystal structure were reported previously,^54^ and the resolved structures were deposited in the crystallographic database (CCDC nos. 2019482 and 2019486). CB6O.Py crystals are characterized by a centrosymmetric triclinic space group, although they crystallize in two polymorphic forms (denoted as α and β) that significantly differ in the organization of molecules and in the unit cell parameters. A characteristic feature of form α is the same orientation of the molecules in the (010) plane that are arranged in parallel rows (Figure 3a,b). The parameters of the unit cell of form α containing one independent molecule in the asymmetric part are as follows (*a*, *b*, *c*, α, β, and γ): 0.577 nm, 1.038 nm, 2.762 nm, 79°, 85°, and 85°. The parallel molecular arrangement in the (010) plane indicates that the molecules within rows are stabilized by direct π–π stacking interactions between the same mesogenic units, i.e., pyrene–pyrene and cyanobiphenyl–cyanobiphenyl. However, the organization along the [010] direction is distinctly different because the orientation of molecules in the subsequent layers is antiparallel. This is clearly visible in the projection of the crystal structure in the (100) plane (Figure 3c). Consequently, the molecular rows in this plane are stabilized by the dominant C–H···π interaction between different pyrene–cyanobiphenyl mesogenic units.

**Figure 4:**
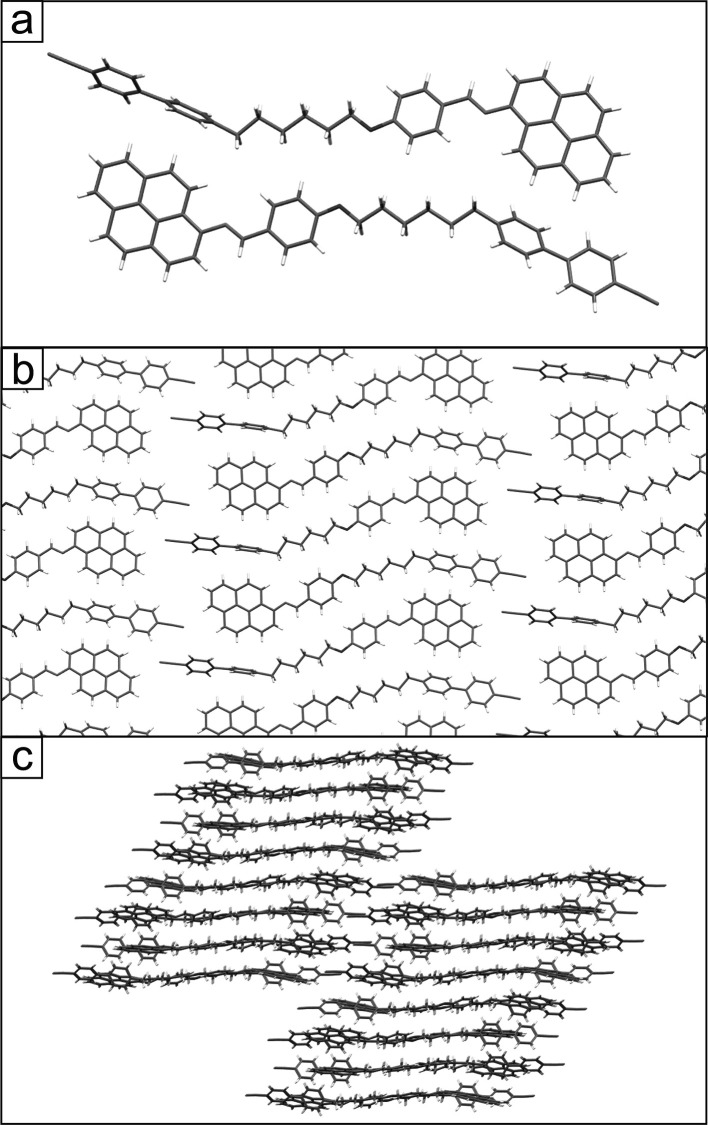
Crystal packing of CB6O.Py in the β polymorph (a, b) in the (010) plane (molecules in rows are oriented antiparallel) and (c) in the (100) plane (from crystallographic database CCDC no. 2019486).

In the case of the second polymorphic form (β), a clearly different arrangement of the molecules practically minimizes the contribution of π–π stacking interactions. In the (010) plane as well as in the (100) one, the molecules in molecular rows are arranged alternately (Figure 4). The unit cell of form β is roughly doubled compared to that of form α and contains four independent molecules in the asymmetric part (*a*, *b*, *c*, α, β, and γ): 1.222 nm, 1.824 nm, 2.860 nm, 89°, 81°, and 86°. The antiparallel arrangement of molecules found in this structure induces strong intercalations of different mesogenic units and reveals the dominant role of the C–H···π interactions in the structure stabilization. To conclude this part of the paper, it can be stated that the CB6O.Py crystals are characterized by a lamellar structure with molecules oriented almost perpendicularly to the plane of the formed layers (Figures 3c and 4c). As a result, the thickness of the layers is slightly smaller than the length of the molecule (corresponding to the (*c*) parameter of the unit cell). At the molecular level, the crystal structure can be considered as a consequence of the competition of two different types of intermolecular interactions. The existence of two polymorphic forms confirms the possibility of the crystal energy minimization through significantly different mutual spatial arrangement of the molecules leading to different distributions and balances of the interactions and packing density.

**Figure 5:**
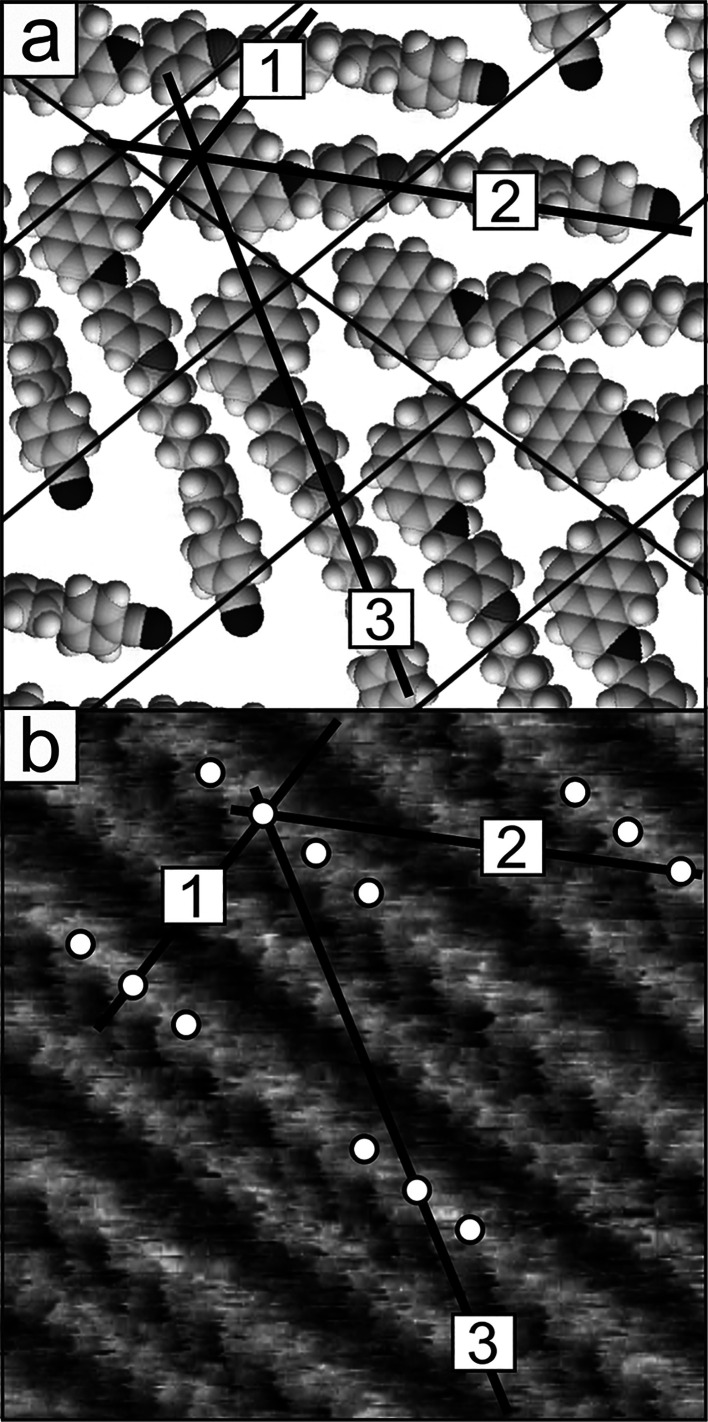
(a) Model of adsorption geometry and (b) corresponding STM image of the CB6O.Py monolayer on HOPG (structure A) showing correlation between molecular arrangement in the monolayer and crystal axes of the graphite substrate surface (marked by additional black lines and numbers 1–3). Scanning area and parameters: (b) 15 × 15 nm^2^, Δ*z*_max_ = 0.48 nm, *I_t_* = 1 nA, and *V*_tip_ = 0.8 V.

Before comparing the ordering of CB6O.Py in a 3D crystal and in a 2D monolayer, it should be recalled that the self-assembly process in both cases occurs under very different conditions. The discussion presented above indicates that the crystal energy minimization forces a strong spatial arrangement of the molecules with the occurrence of different molecular orientations and rotations of the mesogenic units: pyrene as well as phenyl rings in the cyanobiphenyl unit. In the case of the formation of a monomolecular layer, a natural tendency to locate all specific units of the molecule in one plane should be expected. In light of the 3D structure analysis, this tendency is of significant importance. It is a consequence of two factors: (i) termination of the adsorbate above the monolayer and (ii) interactions of the adsorbate with the HOPG substrate, which can induce the entire molecule orientation change within the layer or its individual parts. A comparison of the 2D and 3D structures indicates that the ordering of the molecules in the monolayer shows closer similarity to the ordering in the (010) plane of the crystal structure of the α polymorph than to the β polymorph (compare Figures 2c, 3a, and 4a). In both cases (2D and 3D), the molecules in a single row are parallelly oriented in the same direction. However, there is a fundamental difference in both organizations, as manifested by unit cells of different dimensions. In the case of the 2D structure, it is revealed by different contrast and internal structures of the neighboring bright bands (Figure 2a,b), well observed in the STM images of the monolayer. These differences can only be explained by the fact that the neighboring bands correspond to the areas of localization of different mesogenic units. Therefore, in contrast to the parallel arrangement of molecules in the (010) plane of the 3D structure (Figure 3b), the monolayer is characterized by the proposed herringbone structure with different orientations of molecules from adjacent rows. When these differences are discussed, attention should also be paid to the influence of the substrate on the organization of molecules in the monolayer. In the case of the investigated dimers, two facts are important. First, linear saturated hydrocarbons, when adsorbed on a HOPG surface, have a strong tendency to adopt the *trans–trans* conformation and to orient along the crystal axis of the substrate.^62−66^ Consequently, it should be expected that the alkoxy linker of CB6O.Py should force the orientation of the molecule along one of these axes. Second, a large aromatic part of the molecule (pyrene), when adsorbed flat on the graphite surface, should also adopt an orientation consistent with the surface symmetry axes of the substrate. A similar arrangement was also found in 2D supramolecular organization of some organic semiconductors on HOPG, e.g., flavanthrone derivatives, as previously reported.^66^ In the proposed monolayer structure, the above specific features are taken into consideration. Thus, the molecules from two adjacent rows are rotated by 60° with respect to each other (see Figure 5a). As a consequence, both the alkoxy linkers and the pyrene units are oriented along the graphite surface symmetry axes, as schematically marked in Figure 5 by black lines 1–3. A significant impact of the substrate crystallographic orientation on the monolayer structure is also observed in a much larger range order. In Figure 5b, the periodicity of the internal structure of the brighter bands (R1) is marked with white circles. The positions of these areas along three crystallographic axes of graphite are the same in the consecutive molecular rows. This finding clearly confirms higher-order commensurability, i.e., a close correlation of the periodicity of the monolayer structure with the organization of carbon atoms of the graphite substrate.

**Figure 6:**
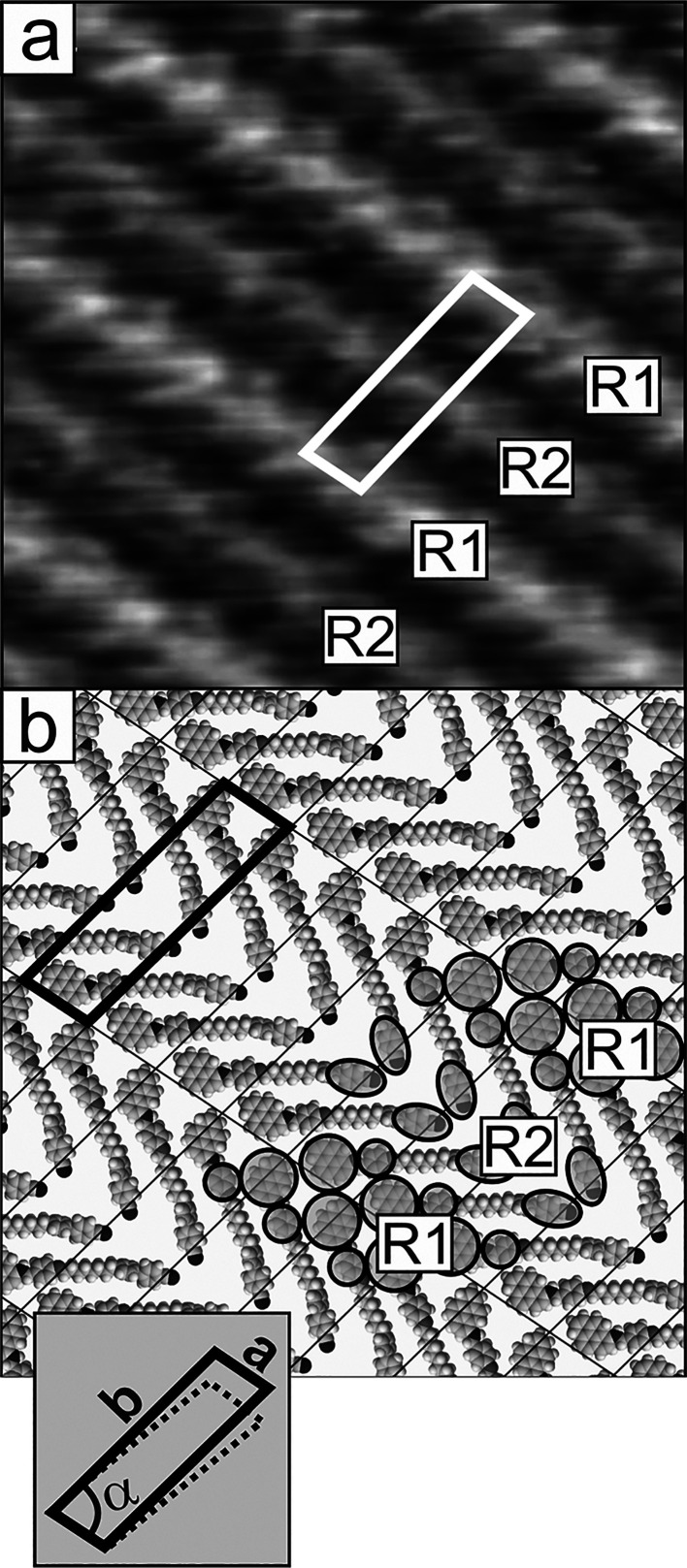
(a) STM image and (b) corresponding model of adsorption geometry of the CB8O.Py monolayer on HOPG (structure A). Scanning area and parameters: (a) 15 × 15 nm^2^, Δ*z*_max_ = 0.4 nm, *I_t_* = 1 nA, and *V*_tip_ = 0.85 V.

For a better understanding of the monolayer structure, the study was extended to a derivative containing two additional methylene groups in its alkoxy linker, namely, CB8O.Py. Figure 6 shows a representative STM image of the CB8O.Py monolayer together with the proposed model of its supramolecular organization. The parameters of the unit cell determined from the STM image are as follows (*a*, *b*, and α): 1.45 ± 0.1 nm, 5.0 ± 0.15 nm, and 78 ± 2°. Comparative analysis with the above-discussed CB6O.Py monolayer indicates a qualitatively high similarity of the 2D order of both derivatives. It should be noted that the shorter dimension of unit cell (*a*) is preserved. In turn, the most significant consequence of linker elongation is an increase in the longer unit cell dimension (*b*). This difference of ca. 0.5 nm is justified by two factors: (i) the extension of the linker length by two methylene groups in CB8O.Py and (ii) herringbone organization in the monolayer of this compound, in which two molecules contribute to this dimension of the unit cell. The presented comparison also indicates that the dominant interaction stabilizing the order of the dimers in rows is the interaction of pyrene groups. The dimension (*a*) of the unit cell does not change going from CB6O.Py to CB8O.Py because the pyrene groups are equally strictly ordered regardless of the linker length. Thus, the dimensions of this large aromatic mesogenic part of the molecule determine the packing density in the row.

At this point, it should be mentioned that the previous XRD studies of CB8O.Py single crystals revealed lamellar structures (CCDC nos. 2019483 and 2019484). The crystals, similar to the above-described CB6O.Py derivative, are characterized by a centrosymmetric triclinic space group with the possibility of forming two polymorphs (denoted as α and β). The unit cell parameters for the longer derivative are as follows (*a*, *b*, *c*, α, β, and γ): 0.573 nm, 1.046 nm, 2.889 nm, 83°, 88°, and 86° (form α); (*a*, *b*, *c*, α, β, γ): 1.157 nm, 2.583 nm, 2.814 nm, 82°, 89°, and 88° (form β). A comparison of the unit cell parameters of the α polymorphs of CB8O.Py and CB6O.Py clearly indicates their similarity. This feature seems to indicate that the expansion of the molecule size through linker elongation leads to distinctly different supramolecular organizations in the 3D crystal and the 2D monolayer. In the former, this arrangement is realized in the bulk whereas in the latter in the plane by an increase in the unit cell dimension in one direction only.

### Structure B (Aggregate Structure)

**Figure 7:**
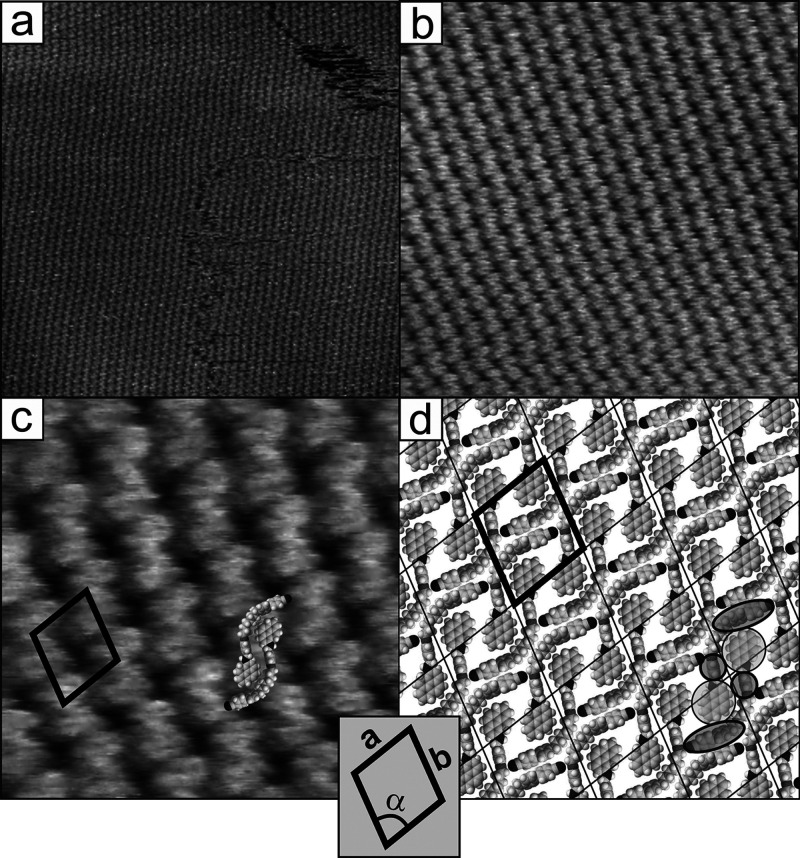
(a–c) STM images and (d) corresponding model of adsorption geometry of the CB8O.Py monolayer on HOPG (structure B). Scanning area and parameters: (a) 98 × 98 nm^2^, Δ*z*_max_ = 0.76 nm, (b) 36 × 36 nm^2^, and Δ*z*_max_ = 0.47 nm; (c) 12 × 12 nm^2^, Δ*z*_max_ = 0.41 nm, *I_t_* = 1 nA, and *V*_tip_ = 0.85 V.

**Figure 8:**
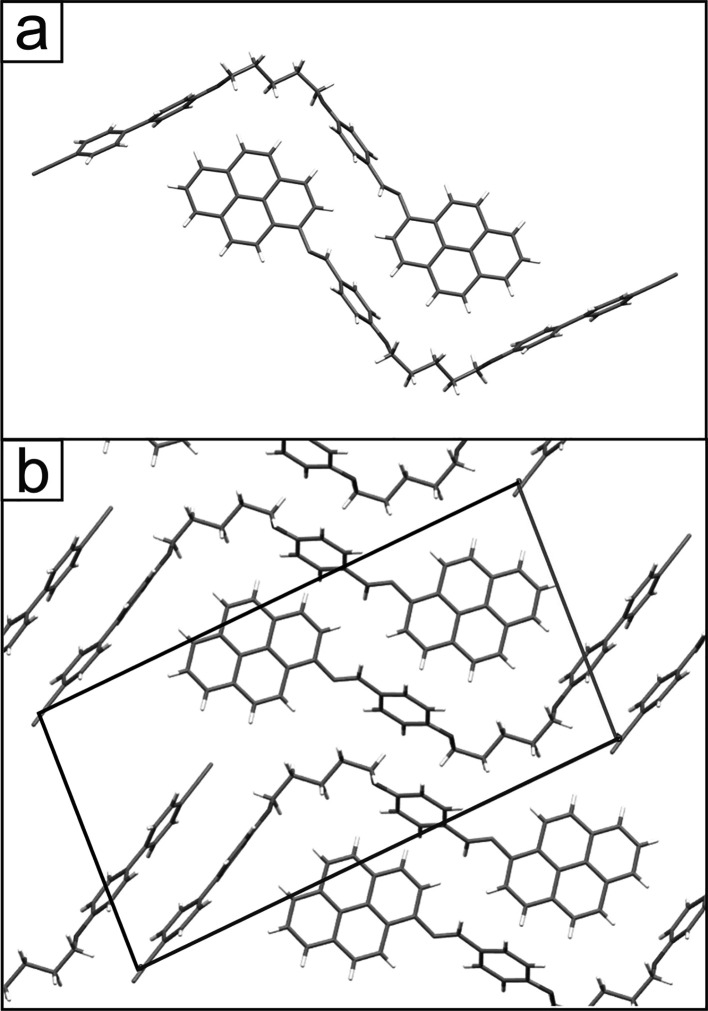
(a, b) Crystal packing of CBO5O.Py in the (010) plane (from crystallographic database CCDC no. 2019485).

The lamellar structure described above is not the only structure found in the monolayers of the discussed compounds. In particular, in the case of CB8O.Py, a completely different organization in the monolayer could also be observed (called the “aggregate structure” in the subsequent text). Figure 7 shows a representative image of the monolayer surface characteristic of the aggregate structure, together with a proposed model of the molecular arrangement. It should be emphasized that systematic statistical analysis of different locations within the image of one particular monolayer, repeated for several dozens of samples, clearly indicates that the aggregate structure is much less frequent than the lamellar one. It gives rise to STM images, which significantly differ from those characteristic of the lamellar structure. The first difference, already seen in low resolved images of larger monolayer areas, is the lack of a difference in the brightness and internal structure of the neighboring rows. The images of higher magnification show another difference. This is a motif of the structure with a characteristic shape, resembling an inverted letter “*z*”, not observed in the images of monolayers adopting the lamellar structure (compare images shown in Figures 7c and 6a for the aggregate and lamellar structures, respectively). This different supramolecular arrangement results in different unit cell parameters, which are (*a*, *b*, α): 2.1 ± 0.15 nm, 2.4 ± 0.15 nm, and 76 ± 2°, as determined from the STM images. This cell is therefore almost rhombic and thus not as elongated as that determined for the lamellar structure. For a better understanding of STM images of the aggregate structure, an important question must be answered, concerning the arrangement of molecules generating this characteristic motif. The correlation of STM images with the dimensions and shape of the CB8O.Py dimer and its possible arrangements indicates that the inverted “z” motif corresponds to an aggregation of two dimers whose shape is determined by close proximity of the pyrene units with the linker groups sticking out, as schematically shown in Figure 7c. The structure of the monolayer in the case of this organization therefore corresponds to the arrangement of such aggregates in two directions, consistent with the directions of the unit cell axis (Figure 7d). It is important to note that the proposed dimer arrangement in the aggregate structure can also be justified by the X-ray single-crystal studies of the 3D organization in molecules of close chemical similarity to CB8O.Py, namely, the diether-linked CBO5O.Py derivative.^54^ The obtained structure has been deposited in the crystallographic database (CCDC no. 2019485) (Figure 8). To the authors’ knowledge, this type of 3D structure has not been so far found for the CB8O.Py dimer by X-ray investigations. The CBO5O.Py crystal is also characterized by a centrosymmetric triclinic space group and cell parameters (*a*, *b*, *c*, *a*, *b*, and *g*): 1.345 nm, 1.869 nm, 2.697 nm, 74°, 86°, and 89°. The structural arrangement in its (010) plane is of very similar if not analogous type to that observed in STM images of the CB8O.Py monolayer (compare Figures 8 and 7).

**Figure 9:**
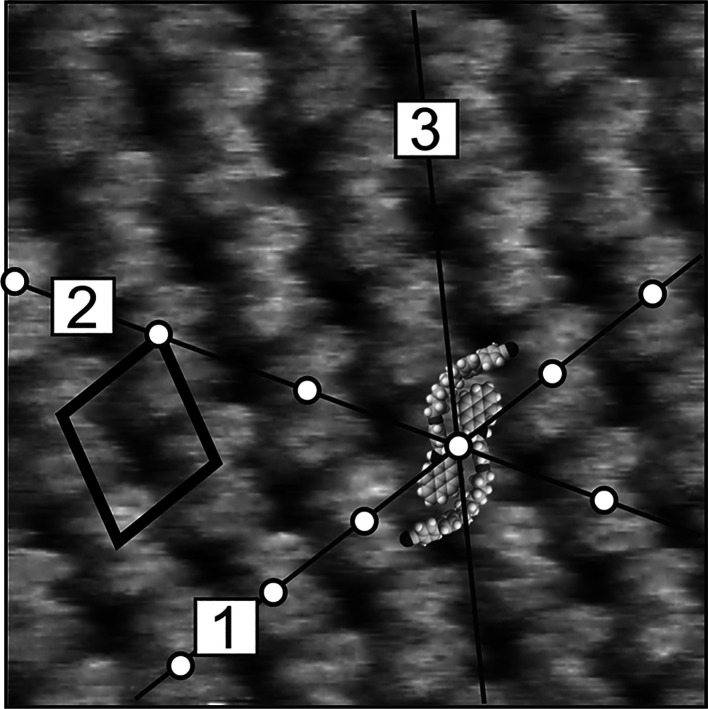
STM image of the CB8O.Py monolayer on HOPG (structure B) with a diagram showing correlation between molecular arrangement in the monolayer and crystal axes of the graphite substrate surface (marked by additional black lines and numbers 1–3). Scanning area and parameters: 12 × 12 nm^2^, Δ*z*_max_ = 0.41 nm, *I_t_* = 1 nA, and *V*_tip_ = 0.85 V.

The motif of the above structure, which is an aggregate of two dimers, is stabilized by the C–H···π interactions between the pyrene unit of one dimer and the phenyl rings of the second dimer, i.e., the one connected to the imine group as well as the biphenyl unit linked to the cyano group (see the scheme of the dimer presented in Figure 9). The aggregate is characterized by two important features that need to be discussed in detail. First, single-crystal XRD studies of the CBO5O.Py derivative confirmed that the orientation of phenyl rings in the linker and in the cyanobiphenyl units of both dimers constituting the aggregate is nearly perpendicular to the plane of the pyrene units. The analysis of the internal structure of this motif in the STM images of the CB8O.Py monolayer (Figure 7) also indicates not planar but rather spatial arrangement of different mesogenic units in both dimers constituting the aggregate. This is reflected by the observed dominance in the contrast of the motif image, i.e., greater brightness of the side areas of the aggregate (upper and lower motif parts in the presented STM image corresponding to the presence of cyanobiphenyl groups and linkers). A lower contribution of the central area corresponding to the location of large fused pyrene units is surprising. The contrast of the image simply reflects a peculiar surface geometry for which the cyanobiphenyl units and phenyl rings are simply rotated and protrude beyond the plane of the monolayer (these areas are schematically marked with darker ellipses in Figure 7d). The second observation is of a more general nature. It should be noted that from the point of view of two-dimensional ordering, the aggregate is characterized by quasi-chiral properties, i.e., in the monolayer, it can adopt two different forms, which are its right-hand and left-hand mirror images. This can be considered as supramolecular aggregation-induced chirality of achiral molecules. The presence of such aggregates not only in monolayers (in the case of CB8O.Py) but also in 3D single crystals (CBO5O.Py) indicates that the formation of this quasi-chiral arrangement is not forced by interactions with the substrate but results from the structural properties of the dimer itself. At the end of this section, the influence of the graphite substrate on the quantitative parameters of this organization should be discussed. The analysis carried out for the lamellar structure also holds in the case of the aggregate structure. The orientation of flat-lying pyrene in the monolayer reflects the crystal axes of the graphite substrate, as schematically presented in Figure 9 by lines 1–3. It should be noted that the location of the structural motif (marked in this image with white circles) in the subsequent rows is analogous along the two graphite axes (marked with 1 and 2). The above observation clearly confirms, as in the case of the lamellar structure, the influence of the carbon atom ordering in the graphite substrate on the aggregate supramolecular 2D organization of CB8O.Py. These interactions may therefore be responsible for the observed quantitative differences in the parameters of the unit cells of the CB8O.Py monolayer and the two-dimensional ordering of CBO5O.Py in the (010) plane of its single crystal. One should be aware of the fact that the above comparison embraces two compounds of very similar chemical nature, albeit containing slightly different linkers; thus, this parameter may also contribute to the observed difference.

### Structure C (Twist-Bend Structure)

**Figure 10:**
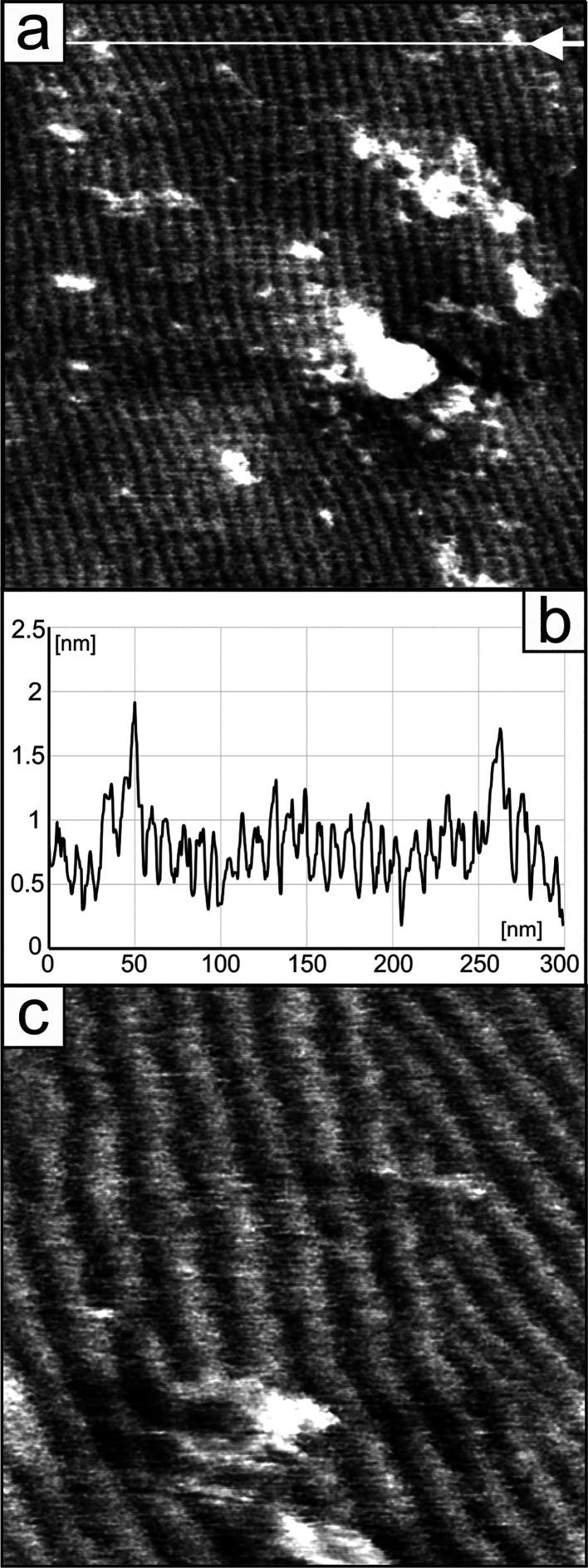
(a, c) AFM images and (b) corresponding line profile (marked in image (a) by a white line and arrow) of the CB8O.Py layer deposited on HOPG (twist-bend nematic phase). Scanning area: (a) 300 × 300 nm^2^ and (c) 100 × 100 nm^2^.

The heliconical twist-bend phase is formed by dimers of the studied CB*n*O.Py family with a linker containing an odd number of segments. Let us recall that in the case of an alkoxy linker, the oxygen atom is treated as a single segment, so this condition corresponds to an even number of methylene groups in the linker. In this type of compound, the twist-bend phase is stable at room temperature but its formation requires quenching at high cooling rates. It should be emphasized that the applied preparation procedure significantly impedes control of the layer thickness. This makes a significant difference in comparison to the method used previously in the studies of symmetric cyanobiphenyl dimers, which directly formed a twist-bend structure at room temperature in a monolayer drop-cast from a solution.^43^ In the case of symmetrical CB*n*CB dimers, it was therefore possible to obtain monolayers of the twist-bend structure on large substrate areas and conduct high-resolution observations by using STM microscopy. Despite the attempts made so far for the asymmetric CB*n*O.Py dimers described in this work, no homogeneous monolayers of the twist-bend structure of the quality required for high-resolution STM investigations were found. The observations were instead limited to thicker layers using AFM microscopy. Figure 10 shows the corresponding images together with a representative surface profile of the CB8O.Py chiral layer formed on a graphite substrate. This surface exhibits different and much larger periodicity, as described previously for structures A and B. It is clearly characterized by regularly occurring parallel stripes with a height of about 0.5 nm (see the surface profile in Figure 10b). The distance between stripes, after averaging from larger areas containing a dozen of stripes in several different places, is in the range of 8.9–9.16 nm. The presented results are in qualitative agreement with previously published observations of the helical twist-bend phase of other compounds of this type performed by means of microscopic techniques TEM and AFM.^67−70^ According to the accepted interpretation, the occurrence of parallel stripes in the layer is a result of parallel arrangement of the helices in the direction perpendicular to the stripes' orientation. The observed periodicity corresponds to periodically occurring differences in the layer thickness as a consequence of changes in the orientation of molecules in the formed helices. Thus, the distance between the stripes indicates the pitch length of the helical structure. It is important to add that the above periodicity is not substrate-dependent, showing very similar values for layers deposited on HOPG (8.9–9.16 nm) or mica (8.9–9.1 nm).

## Conclusions

We used scanning tunneling microscopy (STM) to study the two-dimensional self-organization of asymmetric liquid crystal dimers containing two different mesogenic units: a cyanobiphenyl group and a pyreneiminomethyl phenoxy one. High-resolution imaging of monolayers of two derivatives differing in the length of their oligomethylene linkers (CB6O.Py and CB8O.Py) allowed for a detailed analysis of the two different types of their 2D supramolecular organization. Comparative analysis with the help of already published XRD data confirmed that both monolayer structures are qualitatively consistent with the types of 3D organizations of these dimers in single crystals. However, there are detailed differences resulting from both different organization conditions in 2D and 3D systems as well as the direct effect of the molecules’ interaction with the graphite substrate. In the case of the first of the described organizations, the lamellar structure, the molecules are directly arranged in characteristic parallel molecular rows. Detailed analysis indicates, however, that the organization in the monolayer described above differs from the molecular arrangements in the two reported polymorphic forms of the 3D structure of these compounds. The described difference indicates that in the monolayer structural arrangement, direct interactions of the mesogenic groups of the same type (pyrene–pyrene or cyanobiphenyl–cyanobiphenyl) are preferred. This arises from the fact that contrary to the 3D organization, there is a natural tendency in the monolayer to locate all parts of the adsorbate in one plane, i.e., two different mesogenic units and the linker. In the case of 3D organization in a single crystal, the distribution of intermolecular interactions is more complex and prone to spatial distribution. Comparative studies in relation to two derivatives differing in the length of the oligomethylene linker have shown the influence of this bridging group on the 2D organization in the monolayer. A rather small extension of the linker from six to eight methylene units does not change the type of ordering but causes an extension of the unit cell in one direction. That finding leads to a conclusion that the lamellar structure is in majority stabilized by ordered rows of larger aromatic part of the dimer, i.e., pyrene units. As a consequence, the change in the linker length does not influence the periodicity along these densely packed rows. Moreover, we have also shown for the CB8O.Py molecule the possibility of the existence of a second type of monolayer organization, termed here as “aggregate structure”. In this case, the monolayer is an ordered net of aggregates of characteristic shape, with each of them consisting of two molecules. To the authors’ knowledge, this type of organization has not yet been observed for this type of compound. However, this structural arrangement is qualitatively consistent with the already described 3D organization derived from X-ray investigations of a slightly different derivative, namely, CBO5O.Py. In the case of both types of 2D supramolecular organizations (lamellar and aggregate), the STM studies confirmed a subtle interference of the graphite substrate interaction on the generated structures of the monolayer, which has an effect on the quantitative parameters of the unit cell.

Moreover, more complex sample preparation with quenching at high cooling rates allowed obtaining thicker layers of a stable twist-bend nematic phase. Atomic force microscopy (AFM) confirmed that the surface of this phase is characterized by regularly occurring parallel stripes. The distance between stripes corresponds to the pitch length of the helical structure.

## Notes

Corrie Imrie passed away suddenly on January 14, 2025. The rest of the authors decided to finish the article and submit it with his name as coauthor. This is our tribute to our friend and master.
